# Estimated Vestibulogram (EVEST) for Effective Vestibular Assessment

**DOI:** 10.1155/2021/8845943

**Published:** 2021-03-02

**Authors:** Maja Striteska, Lukas Skoloudik, Martin Valis, Jan Mejzlik, Katerina Trnkova, Martin Chovanec, Oliver Profant, Viktor Chrobok, Jan Kremlacek

**Affiliations:** ^1^Department of Otorhinolaryngology and Head and Neck Surgery, University Hospital Hradec Kralove, Charles University, Faculty of Medicine in Hradec Kralove, Czech Republic; ^2^Department of Otorhinolaryngology, Charles University in Prague, 3rd Faculty of Medicine, University Hospital Kralovske Vinohrady, Czech Republic; ^3^Department of Neurology, University Hospital Hradec Kralove, Charles University, Faculty of Medicine in Hradec Kralove, Czech Republic; ^4^Department of Auditory Neuroscience, Institute of Experimental Medicine of the Czech Academy of Sciences, Prague, Czech Republic; ^5^Department of Biophysics and Department of Pathological Physiology, Charles University, Faculty of Medicine in Hradec Kralove, Czech Republic

## Abstract

**Background:**

The availability and development of methods testing the vestibuloocular reflex (VOR) brought a broader view into the lateral semicircular canal (L-SCC) function. However, the higher number of evaluated parameters makes more difficult the specialist's diagnose-making process.

**Purpose:**

To provide medical specialists, a new diagnostic-graphic tool, Estimated Vestibulogram- EVEST, enabling a quick and easy-to-read visualization and comparison of the VOR test results within the L-SCC.

**Methods:**

The development of EVEST involved 148 participants, including 49 healthy volunteers (28 female and 21 male) and 99 (58 female and 41 male) patients affected by different degrees of peripheral vestibular deficit. The corresponding L-SCC VOR test results, from patients meeting the diagnostic criteria, were used to create the EVEST.

**Results:**

Based on the test results, we depicted and calculated the EVEST vestibular function asymmetry (VFA) in all the groups. To assess a feasibility of EVEST to describe a vestibular function deficit, we calculated sensitivity and specificity of VFA using a receiver operating characteristic curve (ROC) and compared it to single tests. In all the tests, we determined the cutoff value as the point with the highest sensitivity and specificity. For discrimination of any vestibular deficit, the VFA with cutoff 6.5% was more sensitive (91%) and specific (98%) than single tests. Results showed that EVEST is a beneficial graphic tool for quick multifrequency comparison and diagnosis of different types of the peripheral vestibular loss.

**Conclusions:**

EVEST can help to easily evaluate various types of peripheral vestibular lesion.

## 1. Introduction

The field of otoneurology has greatly improved by the use of new testing tools. For assessing a vestibular function, different testing stimuli can be used, such as mechanical, thermal, acoustical, or vibrational. The responses to stimuli which induce (mechanically or thermally) endolymphatic acceleration can be easily compared between each other. Although different parts of a peripheral vestibular endorgan can be evaluated nowadays, lateral semicircular canal (L-SCC) still remains the most frequently tested part with the widest test choice. Clinicians carrying out vestibular tests usually look for asymmetries of the vestibular function between the two sides [1]. In order to assist the clinicians in data analysis of different test results, we have developed a new graph called an Estimated Vestibulogram (EVEST). The EVEST compares the results from the different hVOR tests and shows relations between evaluated parameters, which makes interpretation of test results easy to clinicians.

**Figure 1:**
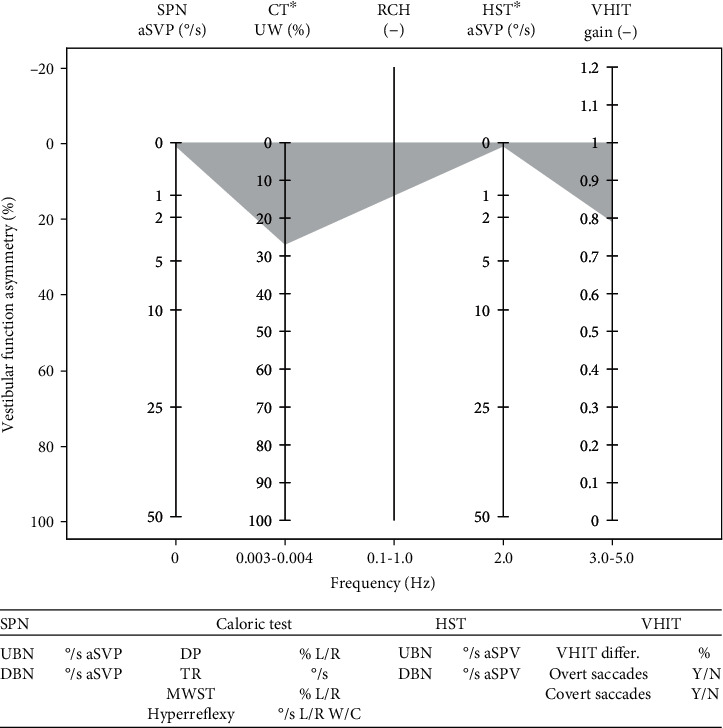
Empty EVEST, normal range in grey [SPN: spontaneous nystagmus; CT: caloric test; RCH: rotatory chair—not applied; HST: head-shaking test; VHIT: video head impulse test; aSPV: average slow phase velocity; MWST: monothermal warm screening test; hyperreflexy of the caloric test according to each lab cutoff values; UBN: upbeating nystagmus; DBN: downbeating nystagmus; DP: directional preponderance of the caloric test; TR: total response of the caloric test; VHIT differ.-: VHIT difference between both sides].

The EVEST has some graphic similarities to pure tone audiogram, which make graph easy to read for the otolaryngologists. The main aim of the EVEST is to provide a graphic overview of the hVOR frequency-specific characteristics in different vestibular lesions or deficits. The aim of this study was to assess the graphic effectiveness of the EVEST to show findings in different vestibular lesions (Figure 1).

## 2. Methods

Data from 148 participants were used to construct EVEST, 49 healthy volunteers (28 female, 21 male, mean age 40.8, SD 10.4), 49 subjects affected by vestibular neuritis (30 females, 19 males, mean age 49, SD 13), and 50 patients with vestibular schwannoma (8 females, 22 males, mean age 54, SD 15).

The inclusion criterion for healthy volunteers was no evidence of any unsteadiness nor vertigo currently or in the past with normal hearing thresholds, for the vestibular schwannoma group was an MRI confirmed diagnosis and for the vestibular neuritis group was an acute unilateral peripheral vestibular lesion without neurological nor cochlear deficit (acute peripheral vestibular deficit confirmed according to HINTS plus protocol [2–4]) and normal MRI result. Participants were examined from January 2016 to July 2019. We tested the feasibility of the EVEST by plotting the different vestibular test results from 3 participants groups in EVEST graphs. We tested the graphic ability of the EVEST to visualize and identify significant vestibular function asymmetry in different vestibular lesions.

### 2.1. Devices Used for a Study

All participants underwent complex neurotologic examination performed by VOG VisualEyes™ 525 (Interacoustics) and VHIT EyeSeeCam (Interacoustics), and examination included measurement of spontaneous nystagmus (SPN), head shaking test (HST), caloric test (CT), VHIT, gaze test, smooth pursuit and saccadic and optokinetic eye movements, cerebellar examination (dystaxia, dysdiadochokinesis, dysmetria, dysartria), evaluation of a gait, grading a truncal ataxia, and skew deviation.

### 2.2. SPN Recording Method

The patient sits upright with visual fixation denied. Tracing for 40 seconds is recorded (sitting position with a head still, goggle closure). If any nystagmus occurs, the VNG software measures its slow phase component.

### 2.3. VOG-Assisted Bithermal Air Caloric Test Procedure

We use warm (50°C) and cold (24°C) air irrigation in each ear for 60 seconds, recording for 120 seconds.

### 2.4. Head-Shaking Test (HST) Procedure

The patient sits upright, visual fixation denied, passive head movements to obtain a frequency of about 2 Hz, amplitude of the head movement 20°, 20 cycles, and then abruptly stopped.

### 2.5. VHIT Procedure

The patient sits upright, with visual fixation of the spot approximately 125 cm distance, unpredictable and passive head turns, peak head velocity is between 150° to 250°/s, and amplitude of head turn 10°–20°.

## 3. Results

### 3.1. Vestibular Neuritis Group

**Figure 2:**
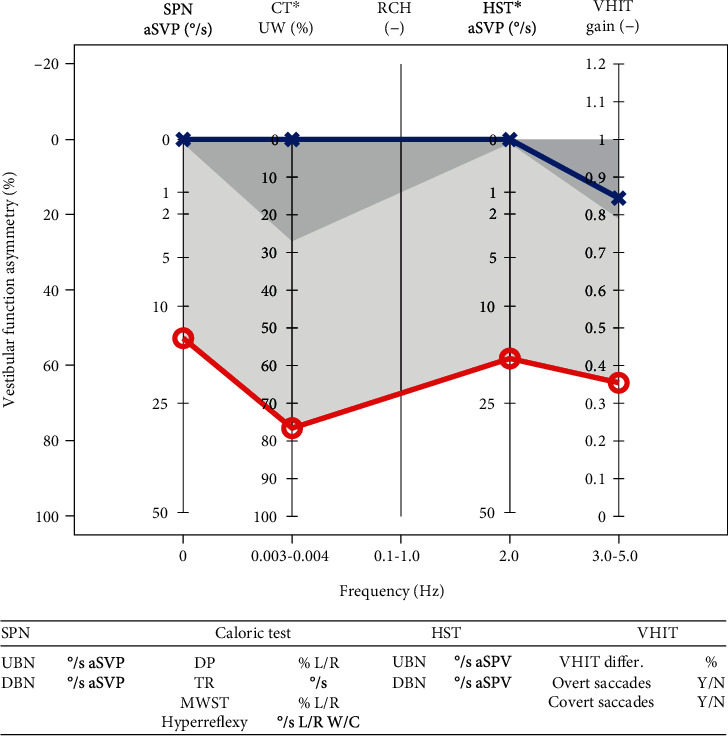
Mean EVEST (mean values were used) for the neuritis group. The right ears are like to be affected, and the left ears are like to be healthy. Note that the multifrequency involvement of all the tests on the affected (red) side and multifrequency significant abnormality of EVEST VFA (61%).

Measured mean spontaneous nystagmus (SPN) was 14°/s average slow phase velocity (aSPV) (SD 5.7, min 5, max 31), and mean head shaking test (HST) was 17.2°/s aSPV (SD 5.3). The mean VHIT on the affected side was 0.35 (SD 0.11) and 0.84 (SD 0.11) on the unaffected side. The caloric test showed mean unilateral weakness (UW) 76.6% (SD 24) on the affected side (Figure 2).

### 3.2. Vestibular Schwannoma Group

**Figure 3:**
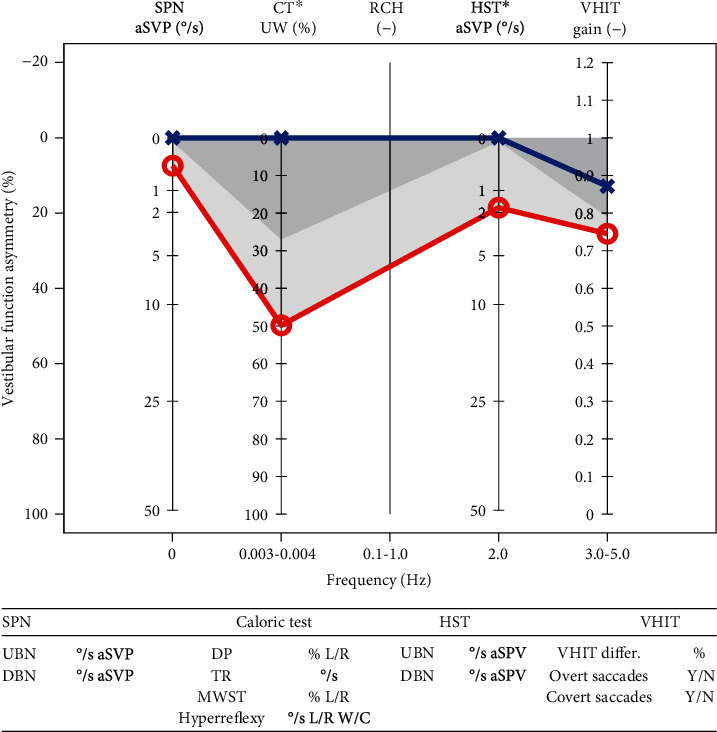
Mean EVEST (mean values were used) for the schwannoma group. The right (red) ears are like to be affected. The caloric test is mostly involved in all Koos schwannoma stages. The EVEST interaural VFA is 20%.

**Figure 4:**
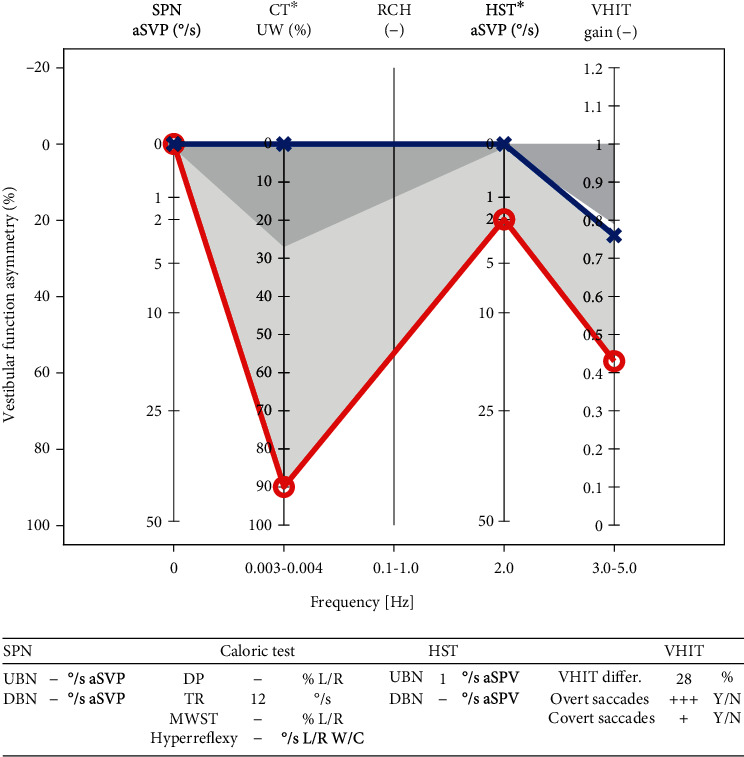
Single patient's EVEST of vestibular schwannoma l.dx., Koos 4 stage. Caloric test and VHIT show severe canal paresis. No spontaneous nystagmus is present, but induced head shaking nystagmus is present. The EVEST interaural VFA is 42%.

Mean SPN was 0.28°/s aSPV (SD 0.53, min 0, max 2), mean HST was 1.76°/s aSPV (SD 2.46), and mean VHIT gain on the affected side was 0.74 (SD 0.21). Mean caloric unilateral weakness on the affected side was 49.8% (SD 32.3). The mean group EVEST visualized a slowly progressing vestibular deficit, detectable in all the patients especially in the caloric test (Figures 3 and 4).

### 3.3. Control Group

**Figure 5:**
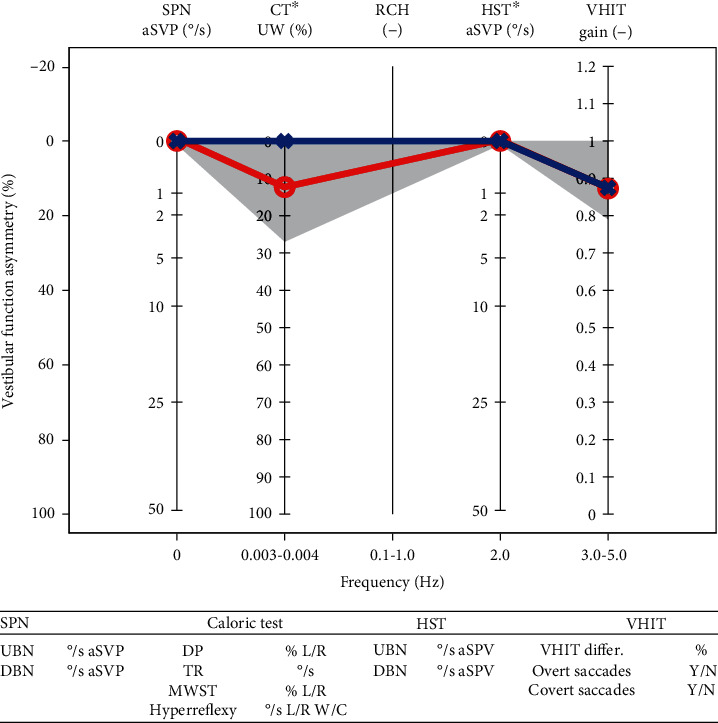
Mean EVEST for the healthy volunteer group. The EVEST interaural VFA is 3%.

No SPN was recorded. No HST was evoked. Unilateral weakness (caloric asymmetry) in the caloric test was within a normal range 0-25% UW (mean 12.7%, SD 7.3). Mean VHIT gain was 0.87 (SD 0.04) (Figure 5).

### 3.4. EVEST Graphic Principles

Different vestibular tests were placed on the horizontal axis according to the tested frequency of the head/endolymph movement. On the vertical axis, there is a vestibular function asymmetry (VFA) between the left and right L-SCC function expressed as a percentage loss (0-100%).

To create the EVEST, we used four mechanical hVOR tests. SPN represents the only hVOR static test with a tested head/endolymph movement frequency of 0 Hz. The caloric test (head-static test which thermally induces acceleration of an endolymphatic flow), HST, and VHIT are hVOR dynamic tests. The caloric test equals the frequency of 0.003–0.004 Hz, HST of 2 Hz, and VHIT of 3-5 Hz. These tests provide complementary insight into the multifrequency hVOR function.

Nowadays, there is still no worldwide agreement on normal and pathologic values for each hVOR test [5]. It is recommended that each department develop their own normative data. We provide our normative data.

The rotatory chair, which is another hVOR dynamic test (tested VOR frequency 0.01–1 Hz), was not used in the construction of the EVEST (both university hospitals included in our study do not perform the rotatory chair tests).

### 3.5. Spontaneous Nystagmus

Nystagmus is an involuntary, rhythmic eye movement with at least one slow phase [6]. The Barány Society made a consensus regarding the different nystagmus patterns [6]. Few studies have focused on the definition of significant spontaneous nystagmus (SPN) without fixation [7–14].

In our healthy group, none of participants had SPN in a single sitting upright and a head still position. Our data are in line with studies showing no evidence of SPN in healthy subjects in the upright sitting position [14–19]. We agree with The British Society of Audiology's recommendation (2015) that a spontaneous nystagmus with fixation removed should be interpreted in light of the overall pattern of the test results and the patient's complaints.

Based on our healthy group data, our reference value for SPN is 0°/s aSPV. The cutoff criteria should be based on the mean ± 2 SD, which is still 0 in our healthy group. Since the cutoff and reference values would be the same, our abnormal cutoff starts at slow phase velocity of 1°/s aSPV.

To display aSPV on a vertical VFA scale, we determined three critical values of 0°/s, 14°/s, and 50°/s with corresponding 0%, 50%, and 100% of VFA. The critical values of aSPV were based on the measured parameters described in our study. The value of 0°/s corresponds to the maximum value in our control group, the SPN value of 14°/s aSPV is the mean, and 50°/s is an augmented SPN maximum value (our maximum was 31°/s) in the neuritis group. The augmentation we made because we presumed the maximum SPN value may be even higher when measured at a vertigo onset (our patients from acute neuritis group were examined at the earliest from 12 hours and later after the vertigo onset). To achieve such mapping, we fitted a power function to these points. The following analytical form fulfilled our constrains: VFA [%] = 14 × aSPV^1/2^ [°/s].

This composition allows detailed insight into the regression of the nystagmus intensity (slowdown of aSPV) regarding vestibular compensation and its time course (initially, fast reduction of SPN intensity in the acute vestibular deficit is followed by the slowed reduction).

The scale on the vertical axis for SPN is an arbitrary composed nonlinear representation of nystagmus slow phase velocity.

Depending on individual labs, the maximum value can be changed, and the *Y* axis may extend to even higher values, if necessary.

To summarize, the SPN axis evaluates the intensity of the spontaneous nystagmus and serves as an arbitrary composed approximative index of the vestibular function loss and functional asymmetry between both sides.

### 3.6. Plotting the Results

We used the average slow phase velocity (aSPV) [°/s] of a detected nystagmus to construct the EVEST. If no spontaneous nystagmus is detected, both ears are plotted as a zero value. If a spontaneous nystagmus is present, we plot its aSPV [°/s] on the vertical axis. The ear from which the spontaneous nystagmus is beating away should be matched on the VFA scale with aSPV measured value [°/s]. Increased SPN (stronger nystagmus intensity) value reflects decrease of a vestibular function in the depicted ear. The remaining ear should be plotted as a zero value. Up or downbeating nystagmus [°/s aSPV] is plotted into the table below the EVEST (see Figure 1).

### 3.7. Caloric Test

The caloric test evaluates the a/symmetry of the caloric responses between the left and right ear and assesses the function of each L-SCC, separately.

If the department's normative data of the caloric responses are not available, the normal limits of both canal paresis and directional preponderance may be taken as ±20% [20].

### 3.8. Plotting the Results

We used unilateral weakness (UW) as a principal result to construct the EVEST [20–23]. There are also directional preponderance (DP), total response (TR), each irrigation response results, and monothermal warm caloric screening test (MWST) that can be used when describing the caloric test results.

In our healthy group, the mean unilateral caloric weakness according to Jongkee's formula [22–24] was 12.7% (SD 7.3%) from which abnormality cutoff was 27.3%. The weaker ear is plotted on the VFA axis with the UW value while the remaining ear is plotted as a zero value.

The monothermal warm screening test (MWST), directional preponderance (DP) and total response (TR) should be written in a table under the EVEST (see Figure 1).

### 3.9. HST

Head-shaking-induced nystagmus is a jerk nystagmus that may follow a prolonged sinusoidal head oscillation [6, 25] In subjects with a dynamic imbalance, a HST-induced nystagmus is often observed, usually beating towards the “better” ear, which decays over about 30 seconds [26]. In general, HST-induced nystagmus is due to a peripheral or central vestibular asymmetry. It should always be interpreted in relation with other data from the EVEST, such as SPN, VHIT, and side of caloric weakness [27].

### 3.10. Plotting the Results

For an EVEST graph, we used the aSPV results [°/s] from head-shaking-induced nystagmus. We did not find any recommendation for normative values and cutoff values in literature for HST-induced nystagmus. In our healthy control group, none had head-shaking-induced nystagmus, or even more than 1-2 nystagmic beats of low amplitude (1-2°/s aSPV) were elucidated. If more than 3 clearly repetitive nystagmus beats after the end of head shake are present, they should be analyzed. In the EVEST, plotting of HST uses the same rules as for an SPN.

### 3.11. VHIT

The head impulse test (HIT) was first described in 1988 by Halmagyi and Curthoys [28]. Implementation of an eye tracking system introduced the routine video head impulse testing (VHIT). The VHIT evaluates the functional state of the all six semicircular canals by measuring the eye movement during an unpredictable head movements [28–32]. In patients with unilateral vestibular lesion, eyes do not compensate for the ipsilesional head turn, and a corrective saccade occurs to return the gaze back to the target [1]. The VHIT calculates the angular VOR gain and visualizes the occurrence of the corrective saccades. Curthoys suggests saccades to be a confirmation of the VHIT gains. And VOR gain to be the most important clinically primary indicator of the vestibular function [1].

### 3.12. Plotting the Results

We used the regression VOR gain value as the principal value for an EVEST. The VOR gain side asymmetry [%] and the presence of the corrective catch-up saccades (overt and covert) are provided in the table below the EVEST. In contrast to other hVOR tests within the EVEST, the VHIT is the only test where measured gains are plotted from both sides, separately.

Mean VHIT gain in our healthy group was 0.87 (SD 0.04). Cutoff values for our healthy group are 0.79-0.95. In the literature, there is still doubt about reference values and cutoffs when assessing VHIT VOR gain [5, 33].

## 4. Discussion

### 4.1. Vestibular Function Asymmetry

To assess a feasibility of EVEST to describe a vestibular function deficit, we introduced a vestibular function asymmetry (VFA) between both sides, expressed as a percentage value. The VFA expresses affected fellow side difference of hVOR deficit. For each side, the VFA is calculated as an average of all results depicted in the EVEST. The EVEST VFA = [14 × SPN^1/2^ [°/s] + CT UW [%] + 14 × HST^1/2^ [°/s] + (1 − VHIT) × 100]/4, while the fellow side is subtracted from the affected, in healthy lower FVA value (side) that should be subtracted from a higher one. To demonstrate the feasibility of EVEST to emphasize patterns of different vestibular lesions, we calculated a mean VFA for each group. In the healthy group, the VFA was 3%; in the vestibular schwannoma, VFA was 20%, and in the neuritis group was 61%.

We calculated sensitivity and specificity of VFA using a receiver operating characteristic curve (ROC) and compared it to single tests. In all the tests, we determined the cutoff value as the point with the highest sensitivity and specificity. In the schwannoma group, EVEST showed higher sensitivity 80% and similar specificity 98% of VFA with cutoff 6.5% comparing to all the tests alone (max sensitivity 70% and specificity 98% in the caloric test, sensitivity 24% in SPN). In the neuritis group, all the tests showed vestibular pathology and therefore, each single test as well as EVEST VFA had the same sensitivity and specificity (100%). For identification of any vestibular deficit, the VFA with cutoff 6.5% was more sensitive (91%) and specific (98%) than single tests (max sensitivity was 84% in caloric and HST, while specificity 98% in caloric, HST, and VHIT). Results showed EVEST benefit to correctly identify diseased individuals as diseased and confirmed an ability of an EVEST to depict a vestibular function deficit.

### 4.2. Limitations of the Study

For EVEST evaluation, we used only two vestibular diagnoses. The true impact on clinical practice will not be known until the other vestibular diagnoses and more patients will be included into the EVEST system. We aim to show the remaining vestibular diagnoses on the EVEST in the next study.

## 5. Conclusions

We are presenting an effective graphical tool to visualize different vestibular deficits. EVEST depicts frequency characteristics of L-SCC from hVOR tests and estimates vestibular function asymmetry (VFA) between both sides. EVEST is an easy-to-read graph, especially for otolaryngologists, who are familiar with graphically similar pure tone audiograms and visualization of a function decrease on a vertical axis and frequency testing on a horizontal axis. We tested feasibility of a graph to visualize a vestibular deficit in two groups with different vestibular lesions. Group with acute vestibular neuritis showed multifrequency hVOR involvement, while the group with vestibular schwannoma had impairment only in some frequencies, which in fact reflects a slow growth of a tumor allowing an ongoing vestibular compensation. Results emerge the need for multifrequency hVOR testing to improve the detection of any vestibular deficit within the L-SCC, which results EVEST serves. The EVEST vestibular function asymmetry (VFA) had higher sensitivity and similar specificity to standalone tests.

EVEST is able to visualize significant vestibular deficits in different vestibular lesions. The EVEST can be also used to visualize patient's follow-up results in order to assess the characteristics of the compensation and reach better conclusions. Future plan is to visualize hVOR-frequency characteristics of the remaining peripheral vestibular lesions (especially paroxysmal diseases) on an EVEST graph.

## Data Availability

Data would be available on request—mail to stritesma@lfhk.cuni.cz.

## Abbreviations

aSPV: Average slow phase velocity
DP: Directional preponderance
EVEST: Estimated Vestibulogram
HST: Head-shaking test
hVOR: Horizontal vestibuloocular reflex
L-SCC: Lateral semicircular canal
MWST: Monothermal caloric warm screening test
SN: Spontaneous nystagmus
TR: Total response
UW: Unilateral weakness
VEMP: Vestibular evoked myogenic potentials
VOR: Vestibuloocular reflex
VOG: Videooculography
VHIT: Video head impulse test
VFA: Vestibular function asymmetry.
